# Health related quality of life assessment in Pakistani paediatric cancer patients using PedsQL^TM^ 4.0 generic core scale and PedsQL™ cancer module

**DOI:** 10.1186/1477-7525-10-52

**Published:** 2012-05-18

**Authors:** Zainab Chaudhry, Salma Siddiqui

**Affiliations:** 1 Clinical Psychology Unit, Government College University, Lahore, 54000, Pakistan; 2 Department of Behaviour Sciences, National University of Science and Technology, Islamabad, Pakistan

**Keywords:** Quality of life, Health related quality of life, Paediatric cancer, Parental perception, Healthy children, Cultural significance

## Abstract

**Background:**

The purpose of the study was to evaluate and compare the HRQOL of paediatric cancer in comparison to the healthy children across age groups, using PedsQL^TM^ 4.0 Generic Core Scales and the PedsQL™ Cancer Module.

**Method:**

The PedsQL^TM^ 4.0 Generic Core Scales and PedsQL Cancer Module 3.0 were administered on 56 children including 26 cancer patients and 30 healthy children while employing self and proxy report forms. Furthermore, the results were compared with their healthy comparison group.

**Results:**

The results indicated a significant relationship between HRQOL reports of cancer patients and their parents. However, the mean of paediatric cancer patients is significantly lower as compare to their healthy comparison group. The mean of proxy report is lower overall on both PedsQL and PedsQL cancer module reports.

**Conclusion:**

Conclusively, overall HRQOL of cancer patients was lower than healthy children but it is quite similar to their parents’ perception. Whereas, the parental mean on PedsQL and PedsQL 3.0 Cancer Module are significantly low. The study indicated a marked difference between cancer patients and healthy children’s HRQOL perception and unfortunately in country like Pakistan where cancer is on increase, no significant work has yet been done to explore this area of research. The present study highlighted the need to focus on the particular psychological health services required to serve the physically challenged population.

## Background

Childhood cancer is accounting for a little part of the world wide cancer encumber. However it becomes more troublesome for source constricted countries like Pakistan, where 60% children die with cancer. This is often because of the late detection and unaffordable treatment of the disease.

Quality of life (QOL) has been described as a subjective term and defined as a person’s sense of social, emotional and physical well-being and his/her ability to function in the ordinary tasks of daily living [1]. Quality of life for children and adolescents cancer patients is a personal, subjective experience, influenced by the individual's internal, immediate, and institutional environments and is most accurately assessed individually while each patient serving as his own standard.

Health related quality of life (HRQOL) is an important outcome of the clinical trials and the populations’ health assessment. It is used interchangeably with the term QOL as it is assumed to be a specific aspect of it. HRQOL is an umbrella term which envelops all the facet of life, not necessarily, only/also acquiescent to health care services.

Health related quality of life (HRQOL) is defined as a patient’s perception of the impact of the disease and treatment functioning in a variety of dimensions including physical, mental and social domains [2]. The concept of HRQOL refers specifically to the impact that health and illness may have on the well-being of an individual, and on patients ability to function in daily life, with respect to physical health, as well as emotional, social and school functioning [3].The concept has also been defined as an individual’s subjective perception of the impact of health status, including disease and treatment, on physical, psychological and social functioning [4]. Although this general definition also applies to HRQOL of children, the specific aspects of a child’s life that comprise these domains are different [4] rated on the same continuum but the factors which define these domains may differ in both the contexts [4,5]. In the case of cancer all these domains are adversely affected, especially for the younger slot of the patient population. QOL for adolescent cancer patients is described as subjective sense of wellbeing during the entire experience of cancer [6].

Although, there are several general and disease specific instruments available for measuring HRQOL, but there are certain issues regarding assessment in the pediatric population [4] including; methodological, proxy and self report issues, use of appropriate age and condition specific assessment tools. In the past years, researches came up with some common domains involved in the general and problem specific measurement tools for HRQOL comprising domains as; physical, social, psychological [7] family interaction, symptoms, usual activities mood, and meaning of being ill [6]. Most of these tools are designed for the adult population [8] and are predominantly designed and used in the source rich societies.

In the recent decade, assessment of general and disease specific HRQOL in paediatric population has amplified greatly, across age groups, disease and populations. Hence, it’s a budding concern to assess the general and disease specific HRQOL of children suffering with chronic health conditions, to acquire a comprehensive evaluation of their condition. It also allows to have a comparison with the healthy children [9].

Due to the comprehensibility, presence of relevant domains and parallel self and parent report forms for the assessment of HRQOL of the paediatric population PedsQL 4.0 Generic Core Scales [2] and PedsQL Cancer Module [10,11] were employed in this study. Both the scales have been validated in different cultures, populations and languages [12-15]. PedsQL 4.0 Generic core scales has been validated and used with healthy children and those suffering with numerous illnesses, as well as distinguish healthy and chronically ill children on the basis of their health status [9,16-18].

The present study is focused upon the investigation and the assessment of HRQOL in Pakistani pediatric cancer patients while employing PedsQL 4.0 Generic core scales and PedsQL 3.0 Cancer Module, as there is no such study, reportedly, conducted in the country which is addressing the issue through any dimension. However there are some studies done on the adult population, but even there the concept of HRQOL is not being addressed.

It is observed that in the source restricted countries like Pakistan, the implications of such concept are quite different from the source rich countries or where the concept has been well emerged and explored.

In this regard, although, it’s a small scale exploratory study, but an initial step towards the investigation of the concept of HRQOL in Pakistani culture, while using a promising assessment tool PedsQL 4.0 and PedsQL 3.0 Cancer Module with paediatric cancer patients and their parents while comparing them with healthy children group. It is hypothesized that the HRQOL of paediatric cancer patients is lower than the healthy children. It is also assumed that HRQOL of paediatric cancer patients and their parents’ perception is affected due to illness, its treatment and its impact.

## Method

The present study was conducted in phases;

Phase I: Translation of the PedsQL^TM^ 4.0 Generic Core Scales and PedsQL Cancer Module

The purpose of the first phase was to translate the **PedsQL**^**TM**^**4.0 Generic Core Scales and PedsQL Cancer Module** into Urdu.

### Sample & procedure

In this phase first, two groups of five bilingual experts were taken for the translation and back translation of scales. The face validity of translation and back translations was determined by a group of experts in the clinical and psychological domains. In order to substantiate the lucidity and utility of the translated tools in our culture, it was tested on five paediatric cancer patients and their parents. The patients were of ages 10-18 yrs including both the genders, 3 males (60%), 2 females (40%) along with their parents (mostly mothers).

Phase II: Validation of the translated scales

It was the pilot phase, semi structured interviews were conducted with the patients and parents. The purpose of these interviews was to provide an opening session to the patients and their parents, before the exploration of the concepts and administration of the translated tools. The interviews were employed to make the concept graspable for the target population as the concepts were novel. The interview in this phase revealed that the responses of the children were fairly similar to the PedsQL items; however there were three items which were identified as different from PedsQL Cancer Module, but were not incorporated in the original scales as it required a separate procedure of validation and standardization.

Phase III: Assessing the HRQOL as perceived by Paediatric cancer patients and their parents

It was the main study phase of the study. The purpose was to assess the HRQOL as perceived by the paediatric cancer patients and their parents.

### Sample & procedure

**Figure 1:**
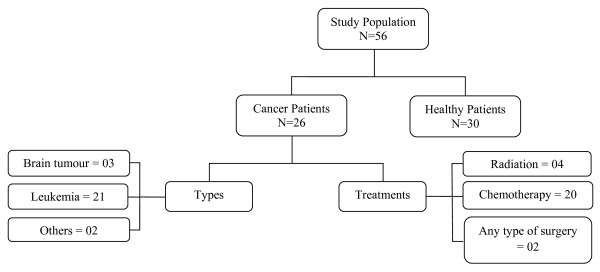
Flow diagram of study population.

The total sample of the study was of 56 children and their parents including 26 cancer patients and a comparison group of 30 healthy children, both with their parents. The sample consisted of both genders of ages 8-18 years, including 14 females (54%) and 12 males (46%) in patient population and 15 females (50%) and 15 males (50%) in the comparison group [Figure 1]. The participants were interviewed and rated their perception of HRQOL on the PedsQL^TM^ 4.0 Generic Core Scales and HRQOL on the PedsQL Cancer Module.

### Instruments

The instruments included in this study are as following:

The PedsQL^TM^ 4.0 Generic Core Scales

The PedsQL generic core scale consists of 23 items and parallel child self-report and parent’s proxy report form for different age groups from 2-18 years. The proxy report forms were designed to get the perception of parents about their child’s HRQOL. The scale has four scales which are physical functioning (8 items), emotional functioning (5 items), social functioning (5 items) and school functioning (5 items). The items are generally similar for all age groups with a slight change in expression for child and parent forms. The PedsQL^TM^ is concise and is a multidimensional scale. In the present study forms for age 8-12 and 13-18 years were used. The items in these scales are scored on a five point Likert scale, ranging from “never a problem” to “almost always a problem” (0 = never a problem; 1 = almost never a problem; 2 = sometimes a problem; 3 = often a problem; 4 = almost always a problem) [9]. Items are reverse-scored and linearly transformed to a 0–100 scale (0 = 100, 1 = 75, 2 = 50, 3 = 25, 4 = 0), so that higher PedsQL 4.0 scores indicate better HRQOL. The PedsQL 4.0 computes the scale scores as well as the Psychosocial health summary score by adding the sum of items responses in the Emotional, Social, and School Functioning Subscales and divide them with the total number of items answered [9].

The PedsQL 3.0 Cancer Module

The PedsQL 3.0 Cancer Module Acute Version is a disease specific instrument and is designed to measure the paediatric cancer specific HRQOL. It has eight domains and 27 items and encompasses both child self report and parent proxy report forms. It has 8 sub-scales including; pain and hurt (2 items), nausea (5 items), procedural anxiety (3 items), treatment anxiety (3 items), worry (3 items), cognitive problems (5 items), perceived physical appearance (3 items), and communication (3 items). The format, instructions, Likert response scale, and scoring method are the same as for PedsQL 4.0 Generic Core Scales. In this scale higher scores indicate better HRQOL [9].

### Demographic variables

The demographic variables included age, gender, type of cancer and duration of illness.

### Sampling strategy

The convenient sampling technique was employed in the study. The paediatric cancer patients were selected from the oncology wards of three government hospitals of Lahore, Pakistan. Firstly, under ethical consideration, the doctors were consulted to medically evaluate the physical condition of the child. The patients with the solid tumours were not included in the sample due to their instable health condition. In this group, 38 paediatric cancer patients were contacted but 26 gave their consent to participate in the study.

In the comparison group, the healthy children and their parents were approached in their homes and public parks. The criterion of their being healthy was not having any serious physical illness or medical condition for the past six months. In this group all the contacted children and parents participated in the study. The consent was obtained from all the participants.

Due to the inclusion of patient population, ethical obligations were considered and the participants had the right to withdraw their participation at any stage of the research activity.

### Statistical analysis

The PedsQL Generic core scale scores for cancer patients were compared with the healthy comparison group across the two age groups (child 8-12 yrs, teenage 13-18 yrs) using independent sample t-test. Pearson product moment correlations were computed to assess the agreement on PedsQL Generic core scale scores between child self-report and parent proxy report, for cancer patients and healthy comparison group as well as relationship between PedsQL Generic core scale scores and PedsQL Cancer module for the cancer patient group was also assessed.

## Results

The PedsQL Generic core scales were completed by 56 children and their parents, including 26 cancer and 30 healthy respondents, along with their parents, shown in Table 1. The PedsQL Cancer module was completed only by the cancer patients and their parents. The study comprised patient population with the mean age of 14 yrs-16.5 yrs and healthy children with the mean age of 10.5 yrs-13 yrs. According to age and gender, there were 10 children (8-12 yrs), 16 teenagers (13-18 yrs), 12 boys (46%), 14 girls (54%) in cancer group , whereas in healthy group there included, 11 children (8-12 yrs) and 19 teenagers (13-18 yrs) having, 15 boys (50%), and 15 girls (50%). The analysis revealed that most of the parents of the paediatric cancer patients were from low SES. The reason was the selection of data from the government hospitals and charity organizations, as they offer diagnosis and treatment of the disease with minimum financial burden on the families.

**Table 1:** Demographic variables for cancer patients and healthy children (n = 56)

**Demographic Variables**	**F**	**%**
Groups		
Cancer patients	26	31.0
Healthy children	30	35.7
Age		
Cancer patients		
8-12 yrs	10	22.2
13-18 yrs	16	35.6
Total	26	57.8
Healthy Children		
8-12 yrs	11	24.4
13-18 yrs	19	42.2
Total	30	66.7
Gender		
Cancer patients		
Male	12	46
Female	14	54
Total	26	
Healthy Children		
Male	15	50
Female	15	50
Total	30	
Diagnosis		
Leukemia 21 21 21	21	80
Brain Tumors	3	11.53
Others	2	7.7
Duration of illness		
6 months	12	46.15
1 year	8	30.76
>1 year	6	23.07
Type of treatment		
Radiation	4	15.9
Chemotherapy	20	76.9
Any type of surgery	2	7.7

### Difference between HRQOL of paediatric cancer patients’ and healthy children (Objective 1)

A significant difference has been assessed between HRQOL of patient population and healthy comparison group, indicated in Table 2, the reason can most evidently be the illness and treatment and its impact on the child’s physical and emotional health.

**Table 2:** Comparison between the PedsQL generic core scale scores of children with cancer and healthy children

**Variables**	**M**	**SD**	**df**	**T**	**P**
**QOL of cancer patients**	46.1154	12.93469	54	-10.989	.000
**QOL of healthy children**	83.1000	12.22871			
**Physical health summary score**					
Cancer patient	42.66	14.78	54	-11.09	.000
Healthy children	84.16	13.20			
**Emotional functioning**					
Cancer patient	42.02	17.06	54	-6.84	.000
Healthy children	74.28	18.00			
**Social functioning**					
Cancer patient	54.42	8.68	54	-9.71	.000
Healthy children	95.66	21.32			
**Psychosocial health summary score**					
Cancer patient	32.91	11.18	54	-15.4	.000
Healthy children	82.21	12.16			

Table indicates the comparison between cancer patients and healthy children on the PedsQL Generic Core scales. The school functioning scale is not included it was not completed by the patient population and thus cannot be compared with the healthy children’s score.

### Relationship between self report and proxy report on paediatric cancer quality of life (Objective 2)

The N = 26 in the cancer patients’ group. Most of the cancer patients were suffering with Acute Lymphoblastic Leukaemia (ALL). The mean duration of their illness was 6 months to 5 years, and the patients were mostly on chemotherapy and mothers were the main respondents for proxy reports (96%) and fathers (4%). Table 3 indicates the mean 46.11 for paediatric patients and 42.07 for the parents. The results in Table 4 indicates a significantly moderate correlation of .64** at **p < 0.01 level of significance between the total score on self and proxy reports.

**Table 3:** Mean and standard deviation of self report and proxy report scores on PedsQL cancer module (N = 26)

**Variables**	**N**	**M**	**SD**
**PedsQL cancer module**			
Self report	26	46.11	13.15
Proxy report	26	42.07	13.17

Table shows mean and standard deviation for the self report and proxy report forms of PedsQL cancer module.

**Table 4:** Correlation between the self report and proxy report scores on the PedsQL cancer module

**Variables**	**1**	**2**
Self report	-	.64**
Proxy report	.64**	

**P = < 0.01 (2-tailed)

Table shows that there is a significant relationship between the HRQOL of pediatric cancer patients and the perception of their parents’ on PedsQL Cancer module.

### Relationship between self report and proxy report on paediatric general quality of life

#### (Objective 3)

The results in the Table 5 indicates that the there is a significantly moderate correlation of .68** on the general quality of life aspect as it is effected due to the illness of the child at **p < 0.01 level of significance between the self and proxy report. Table 6 indicates the inter-correlation between PedsQL 4.0 Generic Core scales and PedsQL 3.0 Cancer module scales for both self and parent proxy reports.

**Table 5:** **Correlation between the self reports and proxy reports on** PedsQL generic core scales total scale scores

**Variables**	**1**	**2**
Self Report	-	.68**
Proxy Report	.68**	-

**P = < 0.01 (2-tailed)

Table shows that there is a significant relationship between the HRQOL of paediatric cancer patients and the perception of the parents about their child’s HRQOL on PedsQL generic core scales.

**Table 6:** Correlation between the PedsQL cancer module and PedsQL 4.0 subscales

**PedsQL cancer module**	**PedsQL 4.0**			
	**Physical health**	**Emotional functioning**	**Social functioning**	**School functioning**
**Child Self Report**				
Pain and hurt	.45*	.22	.23	-
Nausea	.69**	.50**	.43*	-
Procedural anxiety	.46*	.45*	.44*	-
Treatment anxiety	.42*	.46*	.32	-
Worry	.15	.46*	.36	-
Cognitive Problems	.44*	.48*	.49*	-
Perceived Physical Appearance	.44*	-.05	.14	-
Communication	.15	.22	.13	-
**Parents proxy report**				
Pain and hurt	.61**	.18	-.18	-
Nausea	.56**	.25	-.02	-
Procedural anxiety	.48*	.66**	.29	-
Treatment anxiety	.45*	.58**	.21	-
Worry	.51**	.42*	.10	-
Cognitive Problems	-	-	-	-
Perceived Physical Appearance	.42*	.14	.45*	-
Communication	.18	.12	.18	-

*P = < 0.05, **P = < 0.01 (2-tailed).

## Discussion

The purpose of the present study was to assess the HRQOL of paediatric cancer patients and the perception of their parents about the HRQOL of their children as it is compromised by the illness and its treatment, while using PedsQL Generic Core scales and PedsQL Cancer module.

The study in itself has an exploratory aspect. There are a comparatively small number of studies done in Pakistan on the chronically ill patients after the diagnosis of their disease and the effect of their treatment. The researchers are more focused upon the adult assessment of the HRQOL.

Most of the sample was collected from the government hospitals which were usually catering the low socio economic strata of population. It is assessed that the HRQOL of paediatric cancer patients is dependent not only upon the availability of the health services and specialized medical treatment but also upon the quality of the health services provided to them. It was observed that the difference between the quality of health and medical facilities plays a significant role in the HRQOL of the patients, especially, in the resource constricted countries like Pakistan.

The PedsQL^TM^ 4.0 gneric core scales [2] and PedsQL^TM^ 3.0 cancer module [11] were taken from the Mapi Reseach Institute, France [19]. The permission was obtained to use the scales in the research and they helped in understanding and administration of the scales.

While administering a tool on a paediatric cancer patient it is necessary to understand the major domains of the HRQOL. This can assess the extent of specific disease related disabilities in the child as it plays a vital role in altering the health status of the child after the diagnosis of cancer [5]. The domains, which are included in the PedsQL^TM^ 4.0 gneric core scales and PedsQL^TM^ 3.0 cancer module, assess the HRQOL of children on both general and disease specific domains respectively. The assessment was done on both self and proxy reports. In a literature review Varni [18] formulated that using a proxy report in special conditions is recommended but it is not said to be the alternate of the self-report of the children.

The study gives a comparison between the HRQOL of cancer patients and healthy children on PedsQL generic core scales. The results suggested that there is a significant difference between the two groups. The difference is evident on the physical emotional and social domains as well as on psychosocial health summary scores [Table 2. The results positively validated our assumption that there is a different between HRQOL of paediatric cancer patients and healthy children. It is revealed through the study, as done in the other studies that PedsQL Generic Core scales clearly differentiate between patient and healthy population [9,16,17]. The difference between there HRQOL perception was due to their health status. The result does not included the comparison on the school functioning domain as more than 50% of the patients didn’t complete the scale as they were either not going to school for the past 3-6 months or they never attended the school due to the financial constraints. Thus the results for the respective scale for the patient group were not computed. The study as it was first of its nature in the Pakistani health setup and with paediatric patient population, it should be noted here that after the diagnosis of a critical disease like cancer, the financial constraints are so high on the families with low socio economic status. Therefore, along with the prevailing health condition, to save some finances they firstly quit the child’s school as an easy way out. In Pakistan there is no such educational service provided to the ill children during their stay at home or in the hospital.

Spearman’s correlation coefficient between the child self reports and parent-proxy reports on the PedsQL Generic Core Scales and PedsQL Cancer module [Table 6] showed strong positive correlation between both the scales (*P = <0.01*) especially in physical and emotional health. It is assumed that generally to evaluate the physical symptoms of an illness is easier and for the caregivers, perception of HRQOL seem to be more emotion focused.

The Spearman’s correlation coefficient comparisons between the PedsQL 4.0 Generic Core Scales and PedsQL 3.0 Cancer Module suggests that the total scores on the child self reports and parent proxy reports as well as most of the subscales have strong positive correlation. The “Physical health” subscale has high to moderate positive correlation with the “Pain and hurt”, “Nausea”, “Procedural anxiety”, “Treatment anxiety” and “Perceived physical appearance” on both child self reports and parents proxy reports. Whereas the subscale “Nausea” showed high correlation with Emotional and social functioning subscale of PedsQL 4.0 on child self report, similar results have been reported in the reliability and validity study of Japanese version of PedsQL Generic Core scales and PedsQL Cancer module [13]. This suggests that due to physical symptoms like aches and nausea emotional state of the patients is compromised significantly, as they feel low about themselves, thus their social activities also disrupt. Similarly procedural and treatment anxiety subscales have moderate positive correlation with physical emotional and social domains of PedsQL Generic Core scales on child self reports. It can be assumed that an ill child suffering from several physical symptoms can rightly get affected with the frequent hospital visits and medical procedures in all the domains. The significance of these results gets higher in the particular cultural setting as in Pakistan, as having the diagnosis of cancer is a stigma and people get afraid when it comes to the children the situation gets more worse. Generally when the child/adolescent gets the diagnosis of cancer both the parents and the medical staff hide the news from the patient. In this situation when the child/adolescent doesn’t know the reason of frequent hospital visits and painful medical procedures, the anxiety gets elevated. The subscale of “cognitive problems” significantly correlated with the physical, emotional and social domains, whereas “Perceived physical appearance” is positively correlated with physical domain. The “Communication” subscale has no significant correlation with any subscale of PedsQL Generic Core Scale on both child self report and parent proxy report; the reason might be that when the children are not quite ignorant about their actual physical condition then it becomes difficult to communicate their feelings particularly about their health. Overall results of parent proxy reports suggest that the parents perception about their children’s health is more emotion focused and related to their physical health. The reason for this can be assumed to be that physical condition is most evident during illness and its treatment and so the emotional effect of it. Several researches suggest that the childhood cancer had adverse effect on the mental well being of the parents [20-22] and elevate the level of depression and anxiety in the parents of cancer patients, in the early months of the diagnosis [23]. The “School functioning” scale score were not computed for both child self report and parent proxy report.

In the present study, the proxy ratings were mostly based upon the responses of the mothers than the fathers. The reason can be the presence of the mothers as a primary care giver of the child. It was observed that mothers were able to understand the child’s health diligently as compared to fathers. The parents of most of the patients belonged to the low or middle class. The proxy reporting by the mothers at times reveal that the mothers are having the emotion focused coping mechanism which allowed the child and the mother to get affected by the disease in a more emotional manner.

According to the cultural context, an important aspect of the study is that how the medical and treatment expenses are afforded. In the developed societies, the medical expenses are usually afforded by the government or health insurance services. Unfortunately, in our society there is poverty and inequality, which leads to the unavailability of quality health services. The parents of the hospitalized patients reported that there medical expenses were afforded by the hospital, *bait-ul-mal* or with the collaboration of the parents and hospital. This aspect of the health can affect the HRQOL of the child and the parent. However, our study doesn’t clearly depict the effect of SES on the HRQOL’s perception in patients and their parents.

Moreover it was observed that the adolescent subjects were more expressive than the young children of 8-10 years of age either due to language barrier or lack of understanding of the disease and treatment. During the administration of the scales the items were read for most of the children, as they couldn’t read or write, and as well as for the parents, as mostly mothers were also uneducated and initially the concept of quality of life was explained in local language to make it comprehensible. It took 15 minutes to complete for child and parents to complete the scales.

The adolescents who participated in the study were experiencing anger, frustration, emotional turmoil and symptoms of depression. Although there were some who appeared to be composed and tried to adjust with their illness, which was, described as resilience of the adolescents with cancer that in spite of stressful life events some adolescents become adjusted with the challenging life situations [24]. Adolescents who experience emotional turmoil, it is may be due to their altered self-image with which they have to adjust [24]. However, the young children were apparently emotionally stable but appeared to have a sense of uncertainty and confusion may be due to the lack of knowledge about their disease. Another reason described by Woodgate [24] is that children express their symptoms of cancer as feelings and explored that when the physical symptoms of children are approached exclusively as the effect of disease, the children would not describe their real feelings about their illness.

In case of paediatric patients, the assessment of the effect of disease and its treatment on the child has great importance, explained in a literature review. The researcher identified that the HRQOL assessment helps to improve the patient physician communication, increase patient’s satisfaction from the treatment, identify the psychosocial issues, improve the clinical decision making of the health care team according to the patient’s need, and improve the patient’s recovery due to the identification of the problems [25].

The HRQOL researches are the neglected areas in our culture despite its extreme importance in the health care services. These researches help to understand those aspects of health, which affect the health of the patient or a healthy person in an efficient manner.

The present study’s clinical and educational implications are most evident in reference to the cultural background, as the concept of HRQOL is well explored in the western societies. As mentioned earlier, about the small no of researches done in this in Pakistani culture, the primary study is although a small scale exploratory study but provides a basic idea about the concept of HRQOL in the paediatric cancer patients. The implementation of the concept in the health care settings can help to improve the doctor patient relationship, medical decision making about the treatment and its impact, and also patients’ and care givers’ perspective about the disease and its treatment.

The validity and reliability of PedsQL Generic Core scale and the PedsQL Cancer needs to be established in Urdu language in the Pakistani culture, as this study established its feasibility to be used in Pakistan. It can be helpful to establish a counselling protocol after further research in order to assist the children suffering with some chronic illness like cancer to improve their limited social activities according to the health conditions. The study has some limitations including; the study comprised of the small sample due to the limited number of the total population of paediatric cancer patients. Secondly, the results cannot be generalized due to the sampling in which mostly the paediatric cancer patients with ALL were included. And lastly, the illiteracy or the low level of education along with the low socio economic status is some limitation of the sample selection. The reason was that the selected hospitals were mostly catering the low socio economic class.

## Conclusions

In the light of results of the study it is recommended that the concept of HRQOL needs to be understood by the general population, researchers, medical and health care professionals and the patients themselves to make it a global concept. It is required to value the patient’s perception about his illness and alter the treatment plan accordingly. It is highly recommended to conduct qualitative researches with the patient population in order to understand the issues of the patient population especially the paediatric patient population. Health psychologists are required to plan the therapeutic protocols for the children with chronic or terminal illnesses to improve their coping and adjustment with their disease.

## Abbreviation

QOL, Quality of Life; HRQOL, Health Related Quality of Life; WHO, World Health Organization; SES, Socio Economic Status.

## Competing interests

The authors declare that they have no competing interests.

## Authors’ contribution

CZ conceived and conducted the study, did the data collection and writing up the manuscript. SS has contributed in designing the study and helped in analysis and interpretation of the data. The present study is the part of MS in Clinical Psychology degree programme and CZ has worked under the close supervision of SS. All authors read and approved the final manuscript.
